# Distribution of risk factors of hypertension patients in different age groups in Tianjin

**DOI:** 10.1186/s12889-021-10250-9

**Published:** 2021-01-29

**Authors:** Yingyi Zhang, Hua Yang, Min Ren, Ruiying Wang, Fumei Zhao, Ting Liu, Ying Zhang, Zhigang Guo, Hongliang Cong

**Affiliations:** 1 Department of Cardiology, Tianjin Chest Hospital, No. 261 of Taierzhuang South Road, Jinnan District, Tianjin, 300222 China; 2 Tianjin Cardiovascular Institute, Tianjin, 300222 China

**Keywords:** Hypertension, Different age groups, Risk factors, Waist-to-height ratio, Awareness rate

## Abstract

**Background:**

To analyze the risk factors for hypertension in different age groups of urban and rural residents in Tianjin.

**Methods:**

A total of 33,997 people (35–75 years old) from 13 community health service centers and primary hospitals in Tianjin participated in this study. They were divided into the youth group (≤ 40 years old), middle-aged group (41–65 years old), and elderly group (> 65 years old). Then, a questionnaire survey was administered, followed by physical and blood biochemical examinations. The demographic characteristics and prevalence were recorded and counted. Subsequently, risk factors were analyzed using univariate and stepwise multivariate logistic regression analysis.

**Results:**

In the youth, middle-aged, and elderly groups, the prevalence rate of hypertension was 18.65, 51.80, and 76.61%, respectively. Logistic regression analysis showed that obesity(OR: 3.263, 95% CI: 1.039–1.656), men (OR: 2.117, 95% CI: 1.691–2.651), diabetes (OR: 1.978, 95% CI: 1.398–2.799), high triglycerides(OR 1.968 95% CI: 1.590–2.434) and family history of stroke (OR: 1.936, 95% CI: 1.287–2.911) are the five factors in youth. In middle-aged group, the significantly associating factors were obesity (OR: 2.478, 95% CI: 2.330–2.636), diabetes (OR: 2.173, 95% CI: 1.398–2.799), family history of stroke (OR: 1.808, 95% CI: 1.619–2.020), maleness (OR: 1.507, 95% CI: 1.412–1.609),Hypertriglyceridemia (OR 1.490 95% CI: 1.409–1.577),family history of cardiovascular disease (OR: 1.484, 95% CI: 1.307–1.684),Hypercholesterolemia (OR 1.228 95% CI: 1.160–1.299). In the elderly group, obesity (OR: 2.104, 95% CI: 1.830–2.418), family history of strokes (OR: 1.688, 95% CI: 1.243–2.292), diabetes mellitus (OR: 1.544, 95% CI: 1.345–1.773), family history of cardiovascular disease (OR: 1.470, 95% CI: 1.061–2.036), hypertriglyceridemia (OR: 1.348, 95% CI: 1.192–1.524) increased the risk for hypertension. Waist circumference (WC) and waist-to-height ratio (WHtR) increased with age, and the value of these two measures for predicting hypertension was better than BMI in middle-aged group.

**Conclusion:**

Obesity is the most important risk factor for hypertension in all age groups. Diabetes, family history of strokes and high triglyceride were also significant risk factors for all age groups. There was a gender difference between the young and middle-aged groups, with men more likely to hypertension. Waist circumference (WC) and waist-to-height ratio (WHtR) were better predictors of hypertension than BMI in middle-aged group.

## Background

Hypertension is a common chronic disease in the world and an important risk factor for cardiovascular and cerebrovascular diseases,chronic kidney disease and cognitive dysfunction [1, 2]. At the same time, hypertension is one of the single biggest risk factors for death and disability in the global population [3]. At present, the number of hypertension patients in China reaches 270 million. If hypertension is not prevented and controlled in time, it will not only threaten the health of Chinese residents, but also increase the economic burden of the country. As a municipality directly under the Central government with a population of 15 million, Tianjin ranks second only to Beijing in the prevalence rate of hypertension in China [4]. And the incidence of hypertension among young people is increasing year by year, The situation of hypertension prevention and control is grim.

Experience hows that hypertension can be prevented and controlled, and controlling risk factors well can reduce the incidence of hypertension [5, 6]. Understanding the risk factors of hypertension patients is very important for the development of prevention and treatment strategies. Whether there are differences in hypertension among different age groups and whether there are different risk factors is still lacking in in-depth research.

The purpose of this study is to analyze the characteristics and risk factor distribution of hypertension patients in different age groups in Tianjin through large-scale sample study, so as to facilitate the formulation of more targeted preventive measures and improve the prevention and treatment effect.

## Materials and methods

### Patients and grouping

**Fig. 1:**
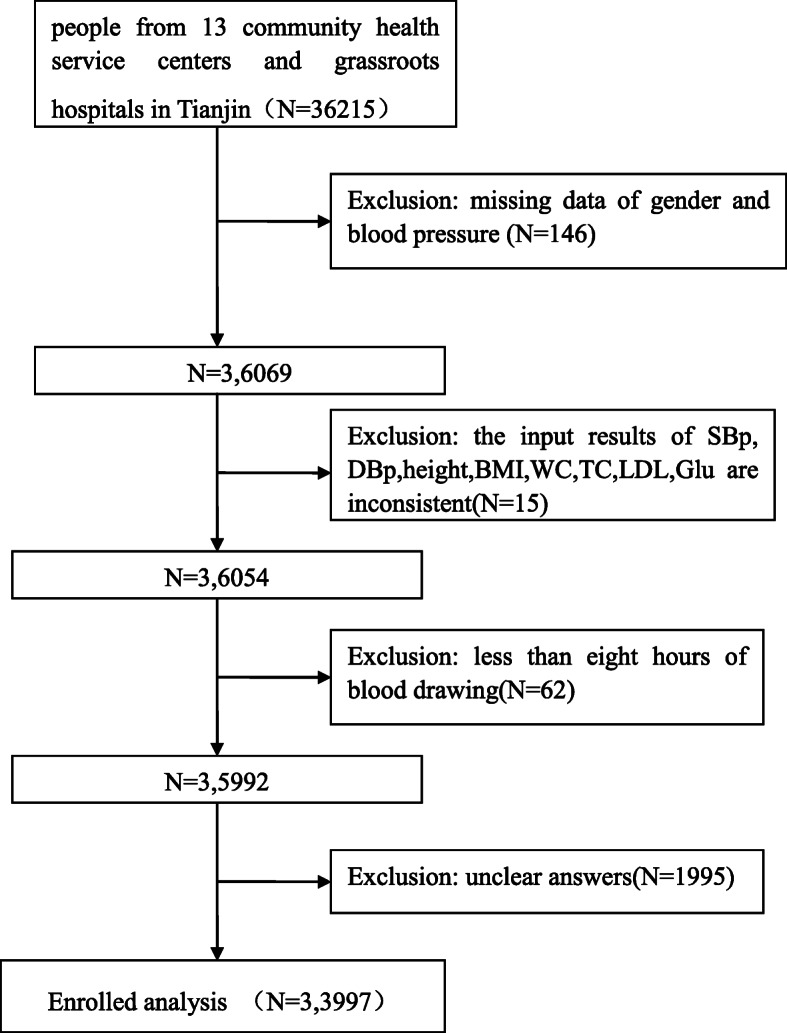
Flow chart of patient inclusion

An investigation conducted in 13 communities / townships in Tianjin from February 2017 to July 2017. A total of 36,215 people from 13 community health service centers and grassroots hospitals in Tianjin participated in this study. Inclusion criteria: (1) age: 35–75 years old (who was born between January 1, 1943 and December 31, 1983); (2) living at the investigation site for more than 6 months in the 12 months prior to screening; (3) participating in this project voluntarily and signing the informed consent. Exclusion criteria: (1) cases with missing data; (2) in order to ensure the accuracy of data entry, the data of height, weight, blood pressure, blood glucose and blood lipid assay were double-entry, i.e., the data were entered twice, and inconsistent data were excluded; (3) Data that respondents answered “unclear”; (4) In order to ensure the accuracy of fasting test value, data of fasting time less than 8 h before blood collection were excluded. Only 33,997 participants were included (Fig. 1). They were divided into the youth group (≤ 40 years old), middle-aged group (41–65 years old), and elderly group (> 65 years old). This study was conducted with approval from the Ethics Committee of Fuwai Hospital of the Chinese Academy of Medical Sciences (2014–574). Written informed consent was obtained from all participants.

### Collection of clinical indicators

The screening consisted of a questionnaire survey and physical and blood biochemical examinations. The questionnaire surveyed demographic characteristics (name, gender, nationality, Identity Card number, current household registration status, current marital status, highest education level, total family income in the past year); personal habits (smoking and drinking; smoking in this study refers to current smokers, and do not include those who have quit smoking. Drinking refers to current drinkers, and do not include abstainers); medical history (hypertension, stroke, diabetes, and myocardial infarction); and family history of cardiovascular disease and stroke. The physical examination covered blood pressure, height, weight, and WC, whereas the blood biochemical examination covered blood glucose and lipid. Electronic height and weight detector are used for height and weight test (brand: Jitai Yongsheng model: GL-150P Origin: South Korea). Upper arm medical electronic sphygmomanometer is used for blood pressure measurement (Brand: Omron MODEL HBP-1300 Origin: Japan). The blood lipids were detected as total cholesterol, triglyceride, HIGH-density lipoprotein and low-density lipoprotein. Blood lipid data were obtained from venous blood collected at the investigation site for rapid blood lipid detection. Testing instruments were produced by CardioChek PA analyzer (Cardigan Biochemical Analyzer), from the United States, CardioTechnology Systems (Polymer Technology Systems). Principle of detection: dry chemical method: reflective photometry. To reduce possible errors in the input process, the above indicators were input twice.

The data source of this study is the screening data of Tianjin area in the major public health service project “early screening and comprehensive intervention for high risk groups of cardiovascular disease” supported by the Ministry of Finance and the national health and Family Planning Commission of China,As the content of China Peace (patient centered evaluation of cardiac events), the screening process and questionnaire content were published in BMJ in 2016 [7].

### Diagnostic criteria and variable definitions

Hypertension: Systolic blood pressure ≥ 140 mmHg, and/or average diastolic blood pressure ≥ 90 mmHg, or history of hypertension [8]. Blood lipid abnormalities [9]: Total cholesterol ≥5.2 mmol/L or 200 mg/dL, high-density lipoprotein cholesterol < 1.0 mmol/L or 40 mg/dL, non-high-density lipoprotein cholesterol ≥4.1 mmol/L or 160 mg/dL, low-density lipoprotein cholesterol ≥3.4 mmol/L or 130 mg/dL, triglyceride ≥1.7 mmol/L or 150 mg/dL. The blood lipids were detected with reflection photometry of dry chemical method by rapid detection from venous blood at the scene of the investigation using the CardioChekPA Analyzer produced by Polymer Technology Systems (PTS) Company of the United States. Diabetes mellitus [10]: Fasting blood glucose ≥7.0 mmol/L (126 mg/ml), or a history of diabetes or of taking hypoglycemic drugs. Obesity [11]: BMI, which equals weight divided by height squared (Kg/m^2^), ≥28 kg/m^2^. Central obesity [12]: WC of male ≥85 cm and WC of female ≥80 cm. WHtR: Waist/height.

### Statistical analysis

SAS 9.2 software was selected for the statistical analysis. The quantitative data were expressed as mean + standard deviation. Kruskal-Wallis test was used for inter-group comparison, and chi-square test was used for classification variable adoption rate or composition ratio analysis. Single-factor logistic regression was adopted to analyze risk factors, and multifactor logistic stepwise regression was used to compare risk factors. The variables included in the multivariate analysis were those with statistically significant differences (*P* < 0.05) in univariate analysis. Correlation analysis was conducted using Spearman’s rank correlation with *P* < 0.05 as the significant difference. SPSS 22.0 was chosen to draw receiver operating characteristic (ROC) curve and area under the curve (AUC), and the cutoff values of BMI, WC, and WHtR were also determined.

## Results

### Demographic characteristics of different age groups

Out of the 33,997 cases, 3008 cases were a part of the youth group (8.85%), which had an average age of 37.82 ± 1.57 years; 24,323 were a part of the middle-aged group (71.55%), which had an average age of 54.14 ± 6.90 years, and 6666 were a part of the elderly group (19.60%), which had an average age of 69.45 ± 2.73 years old. The participants’ demographic characteristics are listed in Table 1. It was obvious that hypertension, diabetes mellitus, cardiovascular disease, stroke, hypercholesterolemia and hypertriglyceridemia had significantly different distributions among the youth, middle-aged, and elderly groups (18.65% vs 51.80% vs 76.61%, *P* < 0.0001; 5.95% vs 18.60% vs 29.60%, *P* < 0.0001; 0.20% vs 2.22% vs 6.68%, *P* < 0.0001; 0.23% vs 3.33% vs 8.52%, *P* < 0.0001; 23.40% vs 43.31% vs 47.67%, *P* < 0.0001, 30.95% vs 39.83% vs 40.04%, *P* < 0.0001,respectively.).

**Table 1:** The demographic characteristics of different age groups

Index	Number (*n* = 33,997, %)	Age group			t /χ^2^value	*P* value
		Youth (*n* = 3008)	Middle-aged (*n* = 24,323, %)	Elderly (*n* = 6666, %)		
Age (years, mean ± SD)	55.69 ± 10.14	37.82 ± 1.57	54.14 ± 6.90	69.45 ± 2.73	21,284.87	< 0.001^*^
Gender						
female	20,907 (61.50)	1873 (62.27)	15,335 (63.05)	3699 (55.49)	127.00	< 0.001^*^
male	13,090 (38.50)	1135 (37.73)	8988 (36.95)	2967 (44.51)		
Nationality						
minority	1326 (3.90)	126 (4.19)	991 (4.07)	209 (3.14)	13.04	0.002^*^
han	32,671 (96.10)	2882 (95.81)	23,332 (95.93)	6457 (96.86)		
Residence						
countryside	8931 (26.27)	747 (24.83)	6663 (27.39)	1521 (22.82)	60.09	< 0.001^*^
town	25,066 (73.73)	2261 (75.17)	17,660 (72.61)	5145 (77.18)		
Marital status						
unmarried	2812 (8.27)	179 (5.95)	1655 (6.80)	978 (14.67)	450.23	< 0.001^*^
married	31,185 (91.73)	2829 (94.05)	22,668 (93.20)	5688 (85.33)		
Education						
primary	6743 (19.83)	163 (5.42)	3883 (15.96)	2697 (40.46)	3773.62	< 0.001^*^
secondary	23,008 (67.68)	1776 (59.04)	17,833 (73.32)	3399 (50.99)		
high	4246 (12.49)	1069 (35.54)	2607 (10.72)	570 (8.55)		
Family annual income (RMB:yuan)						
< 10,000	3009 (8.85)	155 (5.15)	2229 (9.16)	625 (9.38)	321.55	< 0.001^*^
10,000 ~ 50,000	21,157 (62.23)	1569 (52.16)	15,370 (63.19)	4218 (63.28)		
> 50,000	9831 (28.92)	1284 (42.69)	6724 (27.64)	1823 (27.35)		
Smoking						
no	26,100 (76.77)	2443 (81.22)	18,615 (76.53)	5042 (75.64)	38.92	< 0.001^*^
yes	7897 (23.23)	565 (18.78)	5708 (23.47)	1624 (24.36)		
Drinking						
no	24,906 (73.26)	2248 (74.73)	17,547 (72.14)	5111 (76.67)	58.50	< 0.001^*^
yes	9091 (26.74)	760 (25.27)	6776 (27.86)	1555 (23.33)		
Obesity						
no	24,280 (71.42)	2378 (79.06)	17,400 (71.54)	4502 (67.54)	135.32	< 0.001^*^
yes	9717 (28.58)	630 (20.94)	6923 (28.46)	2164 (32.46)		
abdominal obesity						
no	8185 (24.08)	1344 (44.68)	5866 (24.12)	975 (14.63)	1024.30	< 0.001^*^
yes	25,812 (75.92)	1664 (55.32)	18,457 (75.88)	5691 (85.37)		
Hypertension						
no	15,730 (46.27)	2447 (81.35)	11,724 (48.20)	1559 (23.39)	2929.41	< 0.001^*^
yes	18,267 (53.73)	561 (18.65)	12,599 (51.80)	5107 (76.61)		
Diabetes						
no	27,357 (80.47)	2829 (94.05)	19,799 (81.40)	4729 (70.94)	249.65	< 0.001^*^
yes	6640 (19.53)	179 (5.95)	4524 (18.60)	1937 (29.06)		
Cardiovascular disease						
no	33,006 (97.09)	3002 (99.80)	23,783 (97.78)	6221 (93.32)	453.01	< 0.001^*^
yes	991 (2.91)	6 (0.20)	540 (2.22)	445 (6.68)		
Stroke						
no	32,612 (95.93)	3001 (99.77)	23,513 (96.67)	6098 (91.48)	485.32	< 0.001^*^
yes	1385 (4.07)	7 (0.23)	810 (3.33)	568 (8.52)		
Family history of cardiovascular diseases						
no	32,395 (95.29)	2900 (96.41)	23,106 (95.00)	6389 (95.84)	17.63	< 0.001^*^
yes	1602 (4.71)	108 (3.59)	1217 (5.00)	277 (4.16)		
Family history of stroke						
no	31,832 (93.63)	2866 (95.28)	22,648 (93.11)	6318 (94.78)	39.38	< 0.001^*^
yes	2165 (6.37)	142 (4.72)	1675 (6.89)	348 (5.22)		
Hypercholesterolemia						
no	19,580 (57.59)	2304 (76.60)	13,788 (56.69)	3488 (52.33)	528.65	< 0.001^*^
yes	14,417 (42.41)	704 (23.40)	10,535 (43.31)	3178 (47.67)		
Hypertriglyceridemia						
no	20,708 (60.91)	2077 (69.05)	14,634 (60.17)	3997 (59.96)	91.88	< 0.001^*^
yes	13,289 (39.09)	931 (30.95)	9689 (39.83)	2669 (40.04)		

Note: ^*^ indicated *P* < 0.05

### Univariate logistic regression analysis of risk factors for hypertension in different age groups

Univariate logistic regression analysis revealed that in the youth group, maleness (OR: 3.052, 95%CI: 2.527–3.685), smoking (OR: 2.559, 95%CI: 2.079–3.150), drinking (OR: 2.370, 95%CI: 1.951–2.878), obesity (OR: 4.849, 95%CI: 3.972–5.919), central obesity (OR: 4.453, 95%CI: 3.550–5.584), diabetes (OR: 3.389, 95%CI: 2.478–4.635), family history of cardiovascular disease (OR: 1.887, 95%CI: 1.235–2.883), family history of stroke (OR 2.264 95% CI:1.576–3.252),hypercholesterolemia (OR 1.575 95% CI: 1.285–1.931), hypertriglyceridemia (OR 3.308 95% CI: 2.738–3.996)were significantly associated with the occurrence of hypertension. In the middle-aged group, maleness (OR: 1.627, 95%CI: 1.544–1.715), smoking (OR: 1.381, 95%CI: 1.300–1.466), drinking (OR: 1.476, 95%CI: 1.394–1.562), and obesity (OR: 2.890, 95%CI: 2.723–3.067), central obesity (OR: 3.382, 95%CI: 3.175–3.603), diabetes mellitus (OR: 2.624, 95%CI: 2.447–2.813), family history of cardiovascular disease (OR: 1.583, 95%CI: 1.405–1.782), and family history of strokes (OR 1.930 95%CI 1.738–2.143) and, hypercholesterolemia (OR 1.235 95% CI: 1.174–1.299), hypertriglyceridemia (OR 1.884 95% CI: 1.788–1.985), were closely related to hypertension. In the elderly group, high education level (OR: 1.106, 95%CI: 1.009–1.212), high-income level (OR: 1.057, 95%CI: 0.958–1.166), obesity (OR: 2.285, 95%CI: 1.993–2.620), and central obesity (OR: 2.649, 95%CI: 2.295–3.056), diabetes mellitus (OR: 1.726, 95%CI: 1.508–1.977), family history of cardiovascular disease (OR: 1.516, 95%CI: 1.102–2.086), family history of strokes (OR: 1.783, 95% CI: 1.320–2.408) and hypertriglyceridemia (OR 1.535 95% CI: 1.361–1.730),were closely related to hypertension (Table 2).

**Table 2:** Univariate logistic regression analysis of risk factors for hypertension in different age groups

Factor#	Age group								
	Youth			Middle-aged			Elderly		
	OR	95% CI	*P* value	OR	95% CI	*P* value	OR	95% CI	*P* value
Gender	**3.052**	2.527 3.685	< 0.001^*^	**1.627**	1.544 1.715	< 0.001^*^	1.013	0.904 1.136	0.820
Nationality	1.304	0.794 2.143	0.295	**0.773**	0.679 0.879	< 0.001^*^	0.745	0.524 1.060	0.102
Residence	0.894	0.726 1.102	0.294	**0.919**	0.869 0.973	0.004^*^	0.815	0.709 0.937	0.004^*^
Marital status	0.872	0.600 1.268	0.475	1.024	0.927 1.132	0.637	0.936	0.796 1.101	0.426
Education	**0.760**	0.646 0.894	0.001^*^	**0.678**	0.645 0.712	< 0.001^*^	**1.106**	1.009 1.212	0.031^*^
Family annual income	0.865	0.739 1.012	0.069	**0.843**	0.807 0.880	< 0.001^*^	**1.057**	0.958 1.166	0.018^*^
Smoking	**2.559**	2.079 3.150	< 0.001^*^	**1.381**	1.300 1.466	< 0.001^*^	0.699	0.616 0.794	< 0.001^*^
Drinking	**2.370**	1.951 2.878	< 0.001^*^	**1.476**	1.394 1.562	< 0.001^*^	1.067	0.932 1.221	0.349
Obesity	**4.849**	3.972 5.919	< 0.001^*^	**2.890**	2.723 3.067	< 0.001^*^	**2.285**	1.993 2.620	< 0.001^*^
Diabetes	**3.389**	2.478 4.635	< 0.001^*^	**2.624**	2.447 2.813	<.0.001^*^	**1.726**	1.508 1.977	< 0.001^*^
Hypercholesterolemia	1.575	1.285 1.931	<.0.001^*^	1.235	1.174 1.299	< 0.001^*^	0.949	0.847 1.063	0.362
Hypertriglyceridemia	3.308	2.738 3.996	< 0.001^*^	1.884	1.788 1.985	< 0.001^*^	1.535	1.361 1.730	< 0.001^*^
Cardiovascular disease	4.380	0.882 21.758	0.071	**3.774**	3.054 4.664	< 0.001^*^	**1.848**	1.411 2.419	< 0.001^*^
Stroke	3.286	0.733 14.720	0.120	**4.815**	3.998 5.799	< 0.001^*^	**2.929**	2.214 3.875	< 0.001^*^
Family history of cardiovascular diseases	**1.887**	1.235 2.883	0.003^*^	**1.583**	1.405 1.782	< 0.001^*^	**1.516**	1.102 2.086	0.011^*^
Family history of stroke	**2.264**	1.576 3.252	< 0.001^*^	1.930	1.738 2.143	<.0.001^*^	1.783	1.320 2.408	<.0.001^*^

Note: ^*^ indicated *P* < 0.05

# The OR value of the control group is 1.000, gender: female is the control, nationality: minority is the control, residence: city is the control, marital status: unmarried is the control, education: low education level is the control, family income: annual income < 10,000 yuan is the control, for other indicators, the group with an answer of No is taken as control

### Multivariate logistic stepwise regression analysis of risk factors in different age groups

Multivariate stepwise logistic regression analysis was used to conduct correlation analysis on statistically significant factors derived from univariate logistic analysis. The results are shown in Table 3. In the youth group, according to odds ratio (OR) value and standardized regression coefficient, obesity (OR: 3.263, 95% CI: 1.039–1.656)、maleness (OR: 2.117, 95% CI: 1.691–2.651), diabetes (OR: 1.978, 95% CI: 1.398–2.799), Hypertriglyceridemia (OR 1.968 95% CI: 1.590–2.434)、family history of strokes (OR: 1.936, 95% CI: 1.287–2.911) were the top five independent factors influencing hypertension. Further, in middle-aged group, the significantly associating factors were obesity (OR: 2.478, 95% CI: 2.330–2.636), diabetes (OR: 2.173, 95% CI: 1.398–2.799), family history of stroke (OR: 1.808, 95% CI: 1.619–2.020), maleness (OR: 1.507, 95% CI: 1.412–1.609),Hypertriglyceridemia (OR 1.490 95% CI: 1.409–1.577),family history of cardiovascular disease (OR: 1.484, 95% CI: 1.307–1.684),Hypercholesterolemia (OR 1.228 95% CI: 1.160–1.299), drinking (OR: 1.220, 95% CI: 1.139–1.308). Finally, in the elderly group, obesity (OR: 2.104, 95% CI: 1.830–2.418), family history of strokes (OR: 1.688, 95% CI: 1.243–2.292), diabetes mellitus (OR: 1.544, 95% CI: 1.345–1.773), family history of cardiovascular disease (OR: 1.470, 95% CI: 1.061–2.036), hypertriglyceridemia (OR: 1.348, 95% CI: 1.192–1.524) were independently associated with the hypotension.

**Table 3:** Multivariate logistic stepwise regression analysis of risk factors in different age groups

Factor#	Age groups											
	Youth				Middle-aged				Elderly			
	OR	95% CI	Regression coefficient	*P* value	OR	95% CI	Regression coefficient	*P* value	OR	95% CI	Regression coefficient	*P* value
Gender	2.117	1.691 2.651	0.201	< 0.001^*^	1.507	1.412 1.609	0.109	< 0.001^*^	–	–	–	–
Nationality	–	–	–	–	0.818	0.713 0.939	−0.022	0.004^*^	–	–	–	–
Residence	–	–	–	–	1.075	1.009 1.146	0.018	0.026	0.761	0.655 0.884	−0.063	0.001^*^
Education	0.781	0.653 0.934	−0.077	0.007^*^	0.717	0.677 0.759	−0.094	< 0.001^*^	1.159	1.050 1.279	0.051	0.004^*^
Family annual income (RMB:yuan)	–	–	–	–	0.934	0.889 0.981	−0.022	0.007^*^	–	–	–	–
Smoking	–	–	–	–	–	–	–	–	0.774	0.679 0.882	−0.061	< 0.001^*^
Drinking	1.312	1.039 1.656	0.065	0.022^*^	1.220	1.139 1.308	0.049	< 0.001^*^	–	–	–	–
Obesity	3.263	2.633 4.044	0.265	< 0.001^*^	2.478	2.330 2.636	0.226	< 0.001^*^	2.104	1.830 2.418	0.192	< 0.001^*^
Diabetes	1.978	1.398 2.799	0.089	0.001^*^	2.173	2.021 2.337	0.167	< 0.001^*^	1.544	1.345 1.773	<.0.001	0.109
Hypercholesterolemia	1.268	1.008 1.593	0.055	0.042^*^	1.228	1.160 1.299	0.056	< 0.001^*^	–	–	–	–
Hypertriglyceridemia	1.968	1.590 2.434	0.173	< 0.001^*^	1.490	1.409 1.577	0.108	< 0.001^*^	1.348	1.192 1.524	0.081	< 0.001^*^
Family history of cardiovascular diseases	–	–	–	–	1.484	1.307 1.684	0.047	< 0.001^*^	1.470	1.061 2.036	0.042	0.021^*^
Family history of stroke	1.936	1.287 2.911	0.077	0.002^*^	1.808	1.619 2.020	0.083	< 0.001^*^	1.688	1.243 2.292	0.064	0.001^*^

Note: ^*^ indicated *P* < 0.05

# The OR value of the control group is 1.000, gender: female is the control, nationality: minority is the control, residence: city is the control, education: low education level is the control, family income: annual income < 10,000 yuan is the control, for other indicators, the group with an answer of No is taken as control

### Predictive value analysis of BMI, WC, and WHtR to hypertension

**Fig. 2:**
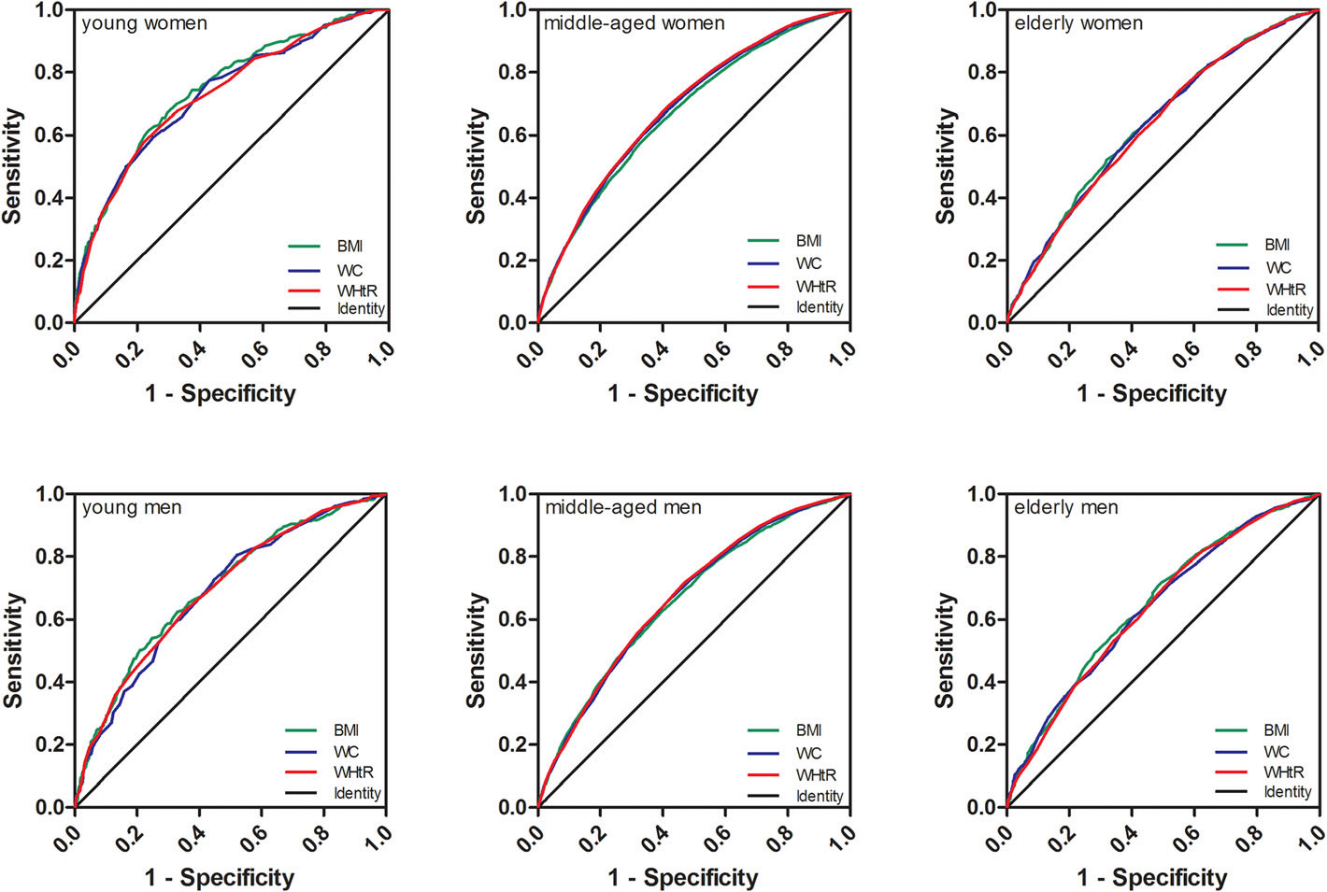
The receiver operating characteristic curve (ROC) curves of female and male drug prescription statistics in different age groups

The ROC curves of BMI, WC, and WHtR for hypertension in different age groups were plotted (Fig. 2), and the cutoff values of different genders were determined. The results indicated that the cutoff values of BMI decreased with increase of age in males. However, the cutoff values increased with age in females. The cutoff values of WC and WHtR increased with age. Last, the AUCs of WC and WHtR were higher than that of BMI shown in Table 4.

**Table 4:** Comparisons of BMI, WC and WHtR among three groups

Fat index	Gender	Age group	Cut-off value	Sensitivity	Specificity	AUC (95% CI)
BMI	male	youth	26.45	0.624	0.671	0.697 (0.664,0.731)
		middle-aged	26.15	0.622	0.607	0.661 (0.650,0.673)
		elderly	25.05	0.708	0.516	0.650 (0.627,0.673)
	female	youth	26.05	0.615	0.765	0.747 (0.712,0.782)
		middle-aged	26.05	0.583	0.665	0.673 (0.664,0.681)
		elderly	26.65	0.521	0.681	0.638 (0.617,0.660)
WC	male	youth	86.9	0.521	0.715	0.686 (0.653,0.719)
		middle-aged	90.1	0.651	0.592	0.665 (0.653,0.676)
		elderly	91.9	0.609	0.596	0.640 (0.617,0.664)
	female	youth	83.5	0.593	0.753	0.731 (0.695,0.768)
		middle-aged	85.45	0.603	0.664	0.685 (0.676,0.693)
		elderly	87.3	0.629	0.568	0.635 (0.614,0.656)
WHtR	male	youth	0.51	0.749	0.530	0.692 (0.659,0.725)
		middle-aged	0.52	0.725	0.524	0.670 (0.658,0.681)
		elderly	0.54	0.667	0.539	0.638 (0.615,0.662)
	female	youth	0.52	0.593	0.775	0.730 (0.695,0.766)
		middle-aged	0.53	0.679	0.596	0.690 (0.682,0.698)
		elderly	0.54	0.745	0.448	0.631 (0.610,0.653)

Abbreviation: *BMI* Body mass index; *WC* Waist circumference; *WHtR* Waist-to-height ratio

## Discussion

Our study revealed that the prevalence rates of hypertension in the three groups were 18.65, 51.80 and 76.61%, respectively. The prevalence of hypertension was higher among middle-aged and elderly people. The prevalence rate was 1/2 to 2/3, consistent with other domestic research results [12, 13]. From 2009 to 2010, data from the United States showed that the awareness rate of hypertension was 81.9%, the treatment rate was 76.6%, and the control rate was 53.3% [14]. The awareness rate, treatment rate and control rate of hypertension are low in China, and the prevention and treatment form of hypertension is not optimistic.

This study included 14 factors that may have an impact on hypertension. Univariate and multivariate logistic regression analysis showed that obesity significantly increased the risk of hypertension in any age group. Especially, it was more significant in the young group. Multi-factor regression analysis showed that the risk of hypertension in the young group was 3.263 times higher than that in the non-obese group, 2.478 and 2.104 times higher than that in the middle and elderly groups, respectively. Framingham study [15]. showed that for every 10% increase in body weight, systolic blood pressure rises by 7 mmHg. Additionally, a study of BMI discovered that the risk of hypertension doubles when the BMI > 23 kg/m^2^, and even when BMI was within the normal range, especially among young people [16, 17]. In Tianjin the prevalence of hypertension is higher than the national average, and more than half of the middle-aged and elderly people with hypertension in this study, Other studies have shown that tianjin residents obesity rates in the country for the first. So, in Tianjin, to strengthen control weight avoid obesity prevention and control of hypertension is the most important factor.

In addition to BMI, there are two indicators of obesity, namely waist circumference and waist ratio, which are more closely related to hypertension, and we did further research. In our study. It can be seen from AUC that AUC of WC and WHtR is higher than BMI in middle-aged group, and abdominal obesity may be a better predictor of hypertension. With age increase, the cut-off point of WC in this study was 86.9–91.9 cm for men and 83.5–87.3 cm for women. The cut-off point was slightly higher, which may be related to the high obesity rate in Tianjin.

This study showed that diabetes was an important risk factor in all three groups, and hypertriglyceridemia was also a risk factor in all three age groups, but hypercholesterolemia was not associated with hypertension in the elderly group. Diabetes mellitus and blood lipid are metabolically related diseases [13] and are closely related to hypertension. Considering the relationship between diabetes mellitus and atherosclerosis induced by insulin resistance [14], blood glucose and lipid should be actively controlled.

Family history of stroke and of cardiovascular disease are closely related to hypertension in middle-aged and elderly patients. Additionally, the results of this study suggested that family history of stroke is a risk factor for hypertension in young people. Therefore, heredity plays an important role in the occurrence of hypertension. Some genetic studies are being carried out on this topic [18]. It is hoped that the occurrence of hypertension can be prevented at the genetic level through new treatment methods.

In young and middle-aged people, maleness, and drinking were risk factors, consistent with previous studies [13]. Although alcohol consumption has a “J-shaped curve” in cardiovascular disease, even a small amount of alcohol consumption in a person with hypertension increases the risk of cardiovascular disease [19]. Therefore, the importance of alcohol cessation should be heavily promoted in this population. Han nationality was the protective factor of the middle-aged group, consistent with the research carried out in Inner Mongolia [20].

There were different conclusions about the protective factors of the elderly group. On the one hand, cities and towns might provide protection for the treatment of hypertension. On the other hand, however, the pressures of life may be greater in cities and towns than in rural areas. Finally, it is worth noting that in the elderly population, high education was the risk factor. These results were different from those of the young and middle-aged population. The reason for this may be that in the young and middle-aged population, high education improved the awareness level and rate of hypertension, thus helping to control the treatment of hypertension [21]. in elderly people with high education, high mental stress and a poor mental state were closely related to hypertension [22]. For this population, in addition to hypertension education, stress education should be provided.

Observations of the influence of smoking on hypertension have proved controversial [23, 24]. In the single-factor regression analysis of this study, the risk of hypertension among smokers in the young and middle-aged groups was significantly increased, but the risk of hypertension was not increased by smoking in the multi-factor regression analysis, and the risk of hypertension among smokers in the elderly group was relatively reduced (OR: 0.774, 95%CI: 0.679 -- 0.882). A number of studies have shown that smoking is not a risk factor for high blood pressure [24, 25], But the risk reduction in older smokers is not well explained, so further research in this area is recommended.

The subjects of this study are 35–75 years old, excluding adults 18–34 years old and over 75 years old, which cannot fully represent the situation of all adults. The number of young people under the age of 40 in the subgroup is significantly lower than that of middle and old age groups, which may influence the conclusion. Dietary habits, dietary structure, exercise frequency and intensity all have an impact on blood pressure. However, there is no detailed investigation of diet and exercise in this study, which is not included in the analysis and discussion. In terms of smoking, smokers in this study were defined as current smokers. There was no more detailed analysis to determine the relationship between quitters and hypertension. The above factors need more detailed investigation data to provide analysis data.

## Conclusion

Obesity is the most important risk factor for hypertension in all age groups. Diabetes, family history of strokes and high triglyceride were also significant risk factors for all age groups. There was a gender difference between the young and middle-aged groups, with men more likely to hypertension. Waist circumference (WC) and waist-to-height ratio (WHtR) were better predictors of hypertension than BMI in middle-aged group.

## Data Availability

We declared that materials described in the manuscript, including all relevant raw data, will be freely available to any scientist wishing to use them for non-commercial purposes, without breaching participant confidentiality. If someone wishes to access the original data, they should contact the corresponding author.

## Abbreviations

WC: Waist circumference
WHtR: Waist-to-height ratio
PTS: Polymer technology systems
ROC: Receiver operating characteristic
AUC: Area under the curve
